# 1761. What Makes Black and Mexican American Children More Vulnerable than White Children to Upper Respiratory Infection?

**DOI:** 10.1093/ofid/ofad500.1592

**Published:** 2023-11-27

**Authors:** Darlene Bhavnani, Peter Dunphy, Elizabeth Matsui

**Affiliations:** Dell Medical School, University of Texas at Austin, Austin, Texas; University of Texas at Austin, Austin, Texas; University of Texas at Austin, Austin, Texas

## Abstract

**Background:**

There is excess risk of upper respiratory infection (URI) among Black and Mexican American children in the US. Evidence suggests that obesity and exposure to nicotine may impair host-pathogen defense mechanisms, increasing susceptibility to URI. Low socioeconomic status (SES) is also linked to a higher risk of URI. Obesity, nicotine exposure, and low SES are more prevalent in racial and ethnic minority populations. We evaluated the extent to which these factors explain the association between race/ethnicity and URI. We also evaluated whether the relationships between these potential risk factors and URI differ by race/ethnicity.

**Methods:**

Children 6-18 years of age from the National Health and Nutritional Examination Survey (2007-2012) were studied. Survey-weighted logistic regression models were used to estimate the association of self-reported cold (URI, defined as cough, cold, phlegm, runny nose, or other respiratory illness (excluding hay fever and allergies) in the past 7 days) to race/ethnicity. Models were adjusted for age and sex. Children with a sex- and age-specific BMI in the 95^th^ percentile or higher were categorized as obese. Serum cotinine levels were used as a biomarker of nicotine exposure. Household SES was analyzed as a summative score which reflected income poverty ratio, home ownership, education, and health insurance.

**Results:**

Black and Mexican American children had 1.34 (95% CI = 1.08-1.67) and 1.53 (95% CI = 1.20- 1.93) times the odds of a URI than White children. Accounting for obesity, serum cotinine, or SES did not attenuate associations between racial and ethnic identity and URI. There were no significant differences in the associations between serum cotinine or SES and URI by race/ethnicity. Associations between obesity and URI were attenuated in Black compared to White children.

**Conclusion:**

Obesity, serum cotinine, and household SES do not help to explain why Black and Mexican American children are at greater risk of a URI than White children. Other contextual factors should be examined to identify the factors that place Black and Mexican American children at greater risk of URI.

**Disclosures:**

**All Authors**: No reported disclosures

